# Distinct characteristics and outcomes in elderly-onset IgA vasculitis (Henoch-Schönlein purpura) with nephritis: Nationwide cohort study of data from the Japan Renal Biopsy Registry (J-RBR)

**DOI:** 10.1371/journal.pone.0196955

**Published:** 2018-05-08

**Authors:** Hiroyuki Komatsu, Shouichi Fujimoto, Shoichi Maruyama, Masashi Mukoyama, Hitoshi Sugiyama, Kazuhiko Tsuruya, Hiroshi Sato, Jun Soma, Junko Yano, Seiji Itano, Tomoya Nishino, Toshinobu Sato, Ichiei Narita, Hitoshi Yokoyama

**Affiliations:** 1 Department of Nephrology, University of Miyazaki Hospital, Miyazaki, Japan; 2 Department of Hemovascular Medicine and Artificial Organs, Faculty of Medicine, University of Miyazaki, Miyazaki, Japan; 3 Department of Nephrology, Nagoya University Graduate School of Medicine, Aichi, Japan; 4 Department of Nephrology, Graduate School of Medical Sciences, Kumamoto University, Kumamoto, Japan; 5 Department of Medicine and Clinical Science, Okayama University Graduate School of Medicine, Dentistry, and Pharmaceutical Science, Okayama, Japan; 6 Department of Integrated Therapy for Chronic Kidney Disease, Graduate School of Medical Sciences, Kyushu University, Fukuoka, Japan; 7 Clinical Pharmacology and Therapeutics, Tohoku University Graduate School of Pharmaceutical Sciences, Sendai, Japan; 8 Department of Nephrology and Rheumatology, Iwate Prefectural Central Hospital, Iwate, Japan; 9 Division of Nephrology, Department of Medicine, Kurume University School of Medicine, Fukuoka, Japan; 10 Division of Nephrology and Hypertension, Department of Internal Medicine, Kawasaki Medical School, Okayama, Japan; 11 Department of Nephrology, Nagasaki University Hospital, Nagasaki, Japan; 12 Department of Nephrology, Sendai Shakai Hoken Hospital, Sendai, Japan; 13 Division of Clinical Nephrology and Rheumatology, Graduate School of Medical and Dental Sciences, Niigata University, Niigata, Japan; 14 Division of Nephrology, Kanazawa Medical University School of Medicine, Ishikawa, Japan; INSERM1163, FRANCE

## Abstract

**Background:**

The clinical presentation and prognosis of adult and elderly patients with IgA vasculitis (Henoch-Schönlein purpura) accompanied by nephritis (IgAV-N) have not been investigated in detail. We therefore surveyed the features and outcomes of IgAV-N based on nationwide data derived from the Japan Renal Biopsy Registry (J-RBR).

**Methods:**

This multi-center cohort study compared the clinicopathological parameters at diagnosis, initial therapies and outcomes between 106 adult (age 19–64 years) and 46 elderly (≥65 years) patients with IgAV-N who were registered in the J-RBR between 2007 and 2012. The primary end-points comprised a 50% increase in serum creatinine (sCr) values or end-stage kidney disease. Factors affecting a decrease in renal function were assessed using Cox proportional hazards models.

**Results:**

Rates of hypertension, impaired renal function, hypoalbuminemia and crescentic glomerulonephritis were significantly higher among the elderly, than the adult patients. About 80% and 60% of the patients in both groups were respectively treated with corticosteroid and a renin-angiotensin system (RAS) blockade. Both groups had favorable renal survival rates for nine years (93.6% and 91.4% of the adult and elderly patients, respectively). Significantly more elderly than adult patients developed a 50% increase in sCr during a mean observation period of 3.9 years (21.7% vs. 4.7%, p = 0.012), and significantly fewer elderly, than adult patients achieved clinical remission (23.9% vs. 46.2%, p = 0.016). Multivariate analysis selected advanced age (≥65 years) and lower serum albumin values as independent prognostic factors for a decline in renal function, whereas steroid pulse therapy helped to preserve renal function.

**Conclusions:**

The renal prognosis of adult and elderly patients with IgAV-N was favorable when treated aggressively with corticosteroid and RAS blockade. However, the course of renal function should be carefully monitored in patients aged over 65 years and those with hypoalbuminemia.

## Introduction

Immunoglobulin A vasculitis (IgAV) is a type of immune complex vasculitis characterized by impaired systemic small vessels. The International Chapel Hill Consensus Conference proposed revising the name Henoch-Schӧnlein purpura (HSP) and definition in 2012 [1,2]. Clinical symptoms are mainly represented by cutaneous purpura, arthritis, gastrointestinal pain/bleeding, and abnormal urinary findings with or without impaired renal function, and about 30%–60% of patients with IgAV develop nephritis (IgAV-N) [3,4]. Although the pathogenic mechanism of IgAV remains unknown, several progressive studies have shown that the onset of IgAV as well as IgA nephropathy (IgAN) might be associated with aberrantly glycosylated IgA1 [5–9].

We previously reported differences and relationships of clinico-pathological findings between IgAV-N (n = 513) and IgAN (n = 5,679) using the Japan Renal Biopsy Registry (J-RBR), a nationwide prospective registry system of renal biopsies [10]. That study uncovered a bimodal age distribution at IgAV-N diagnosis with peaks at the ages of 1–19 and 60–69 years, and more severe clinicopathological findings including blood pressure (BP), proteinuria and acute histological lesions in adult and elderly, than in pediatric patients [10]. However, the cross-sectional study could not determine a relationship between clinicopathological findings at diagnosis and renal outcomes in adult and elderly patients [10]. Several studies also have indicated that histological damage, hypertension and proteinuria > 1g/day are dominant predictors for the progression of pediatric and/or adult patients with IgAV-N [11–16]. Meanwhile, a few studies of adult and elderly patients have investigated the relationship between prognosis and initial treatment [9, 17–19].

We therefore conducted a multi-center cohort study to clarify prognostic factors as well as renal and life prognoses among adult and elderly patients. We also determined the current status of initial treatment for IgAV-N in Japan, and assessed the effects of treatment modalities on renal prognosis.

## Materials and methods

### Selection of patients from J-RBR

**Fig 1:**
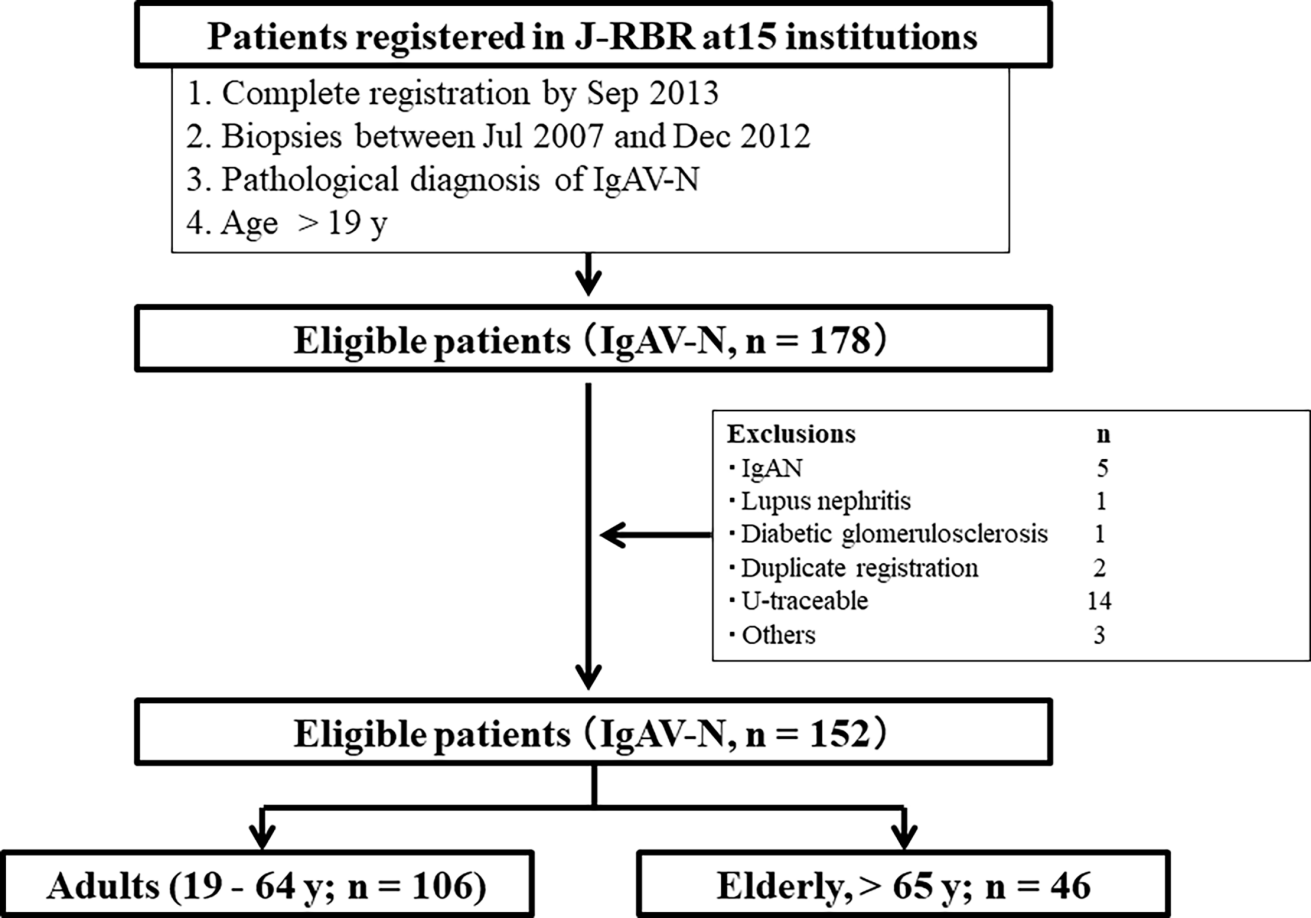
Enrollment and grouping of patients. Among 152 patients who met inclusion criteria, we classified 106 of them as adults (age, 19–64 years), and 46 as elderly (age, ≥ 65 years).

The Japanese Society of Nephrology (JSN) established the J-RBR in 2007 [20, 21], and it is registered under the Clinical Trial Registry of University Hospital Medical Information System (UMIN) in Japan (Registration No: UMIN000000618). Patients’ data from affiliated institutions were registered on the J-RBR website using the Internet Data and Information Center for Medical Research (INDICE) system of UMIN. The main registration data comprised basic information about the patients, date and number of renal biopsies, pathological information based on pathogenesis and histopathology, urinary and blood findings, and coexisting hypertension and diabetes. However, follow-up data to analyze outcomes could not be collected. We therefore planned a new multi-center cohort study of registered patients and institutions [10]. The patients consented to our access to their medical records in the J-RBR, and to participation in this multi-center cohort study. The written informed consent was obtained from all of participants in J-RBR. The consent was obtained from parents if the participant was minor. The Ethics Review Board of the JSN and each research institution for the cohort study approved the present study in accordance with the Declaration of Helsinki (JSN approval number 31; Jun 23, 2016). The research institutions were officially announced on the website of JSN (https://www.jsn.or.jp/). Among 18,967 patients with biopsy-proven disease who were registered in this system between July 2007 and December 2012, 513 diagnosed with IgAV-N based on pathological findings were registered from 64 institutions. We selected 15 institutions that had registered more than five patients aged ≥ 19 years. Among 178 eligible patients, we excluded those with a possibility of a different diagnosis and registration error, as well as those who were untraceable. We finally selected 152 patients who met the inclusion criteria (Fig 1), and allocated them to adult and elderly groups according to being aged 19–64 (n = 106) and ≥ 65 (n = 46) years at the time of the present study.

### Collection of clinical and pathological data from J-RBR database and additional investigations

As described in detail previously [10], the registered basic information, as well as urinary findings, blood findings and BP were assessed in the present study. Estimated glomerular filtration rates (eGFR) were calculated using the modified equation for Japanese [22]. The J-RBR also requires classification based on pathogenesis and histopathology [10]. The histopathology of IgAV-N, which was the pathogenesis of all our selected patients, was evaluated as mesangial proliferative glomerulonephritis, endocapillary proliferative glomerulonephritis, minor glomerular abnormalities, focal segmental glomerulosclerosis, membranous nephropathy, membranoproliferative glomerulonephritis, crescentic and necrotizing glomerulonephritis, and others.

Clinical and pathological data were also collected. The number of biopsied glomeruli, glomeruli with endocapillary proliferative lesions and glomeruli with crescentic lesions were collected as important pathological findings. Oral corticosteroid with the initial dose, steroid pulse therapy with the number of courses, renin-angiotensin system (RAS) blockade, immunosuppressive agents with descriptions, and tonsillectomy were examined as modalities of initial treatment. Almost all urinary and blood items in J-RBR were re-examined during follow-up and/or at the final observation period. The presence or absence of end-stage kidney disease (ESKD) with renal replacement therapy (RRT), death, cardio-vascular disease, malignancy and diabetes mellitus were also surveyed to evaluate prognoses.

### Evaluation of renal outcomes and cardiovascular events

The primary outcome was a decline in renal function as indicated by a 50% increase in sCr from baseline or ESKD with RRT comprising hemodialysis, peritoneal dialysis and renal transplant. Secondary outcomes comprised survival and the onset of cardiovascular events, namely, acute coronary syndrome (myocardial infarction and unstable angina), aortic dissection, intracranial or subarachnoid hemorrhage, cerebral infarction and peripheral artery disease.

Clinical remission (CR) was defined as the disappearance of hematuria and proteinuria. The disappearance of hematuria was defined as findings of < 5/HPF of red blood cells in sediment or (-) ∼ (±) in dipstick tests. The disappearance of proteinuria was also defined as < 0.3 g/day of protein in 24-h urine samples, a UP/UCr ratio of < 0.3 in spot urine or (-) ∼ (±) in dipstick tests. Hypertension was defined as systolic BP > 140 mmHg and/or diastolic BP > 90 mmHg or under treatment with antihypertensive drugs before diagnosis.

### Statistical analysis

Continuous variables are presented as means ± standard deviation (SD). Clinical parameters of the two groups were compared using unpaired t-tests for normally distributed continuous variables, or Mann-Whitney U tests for non-normally distributed continued variables. Differences in proportions were evaluated using χ^2^ independent tests or Fisher exact tests for 2 × 2 tables, depending on the number of categories. Rates of renal survival and of freedom from declining renal function (50% increase sCr from baseline) in the two groups were analyzed using the Kaplan-Meier method, and differences in survival curves were compared using log-rank tests. The impact of multiple covariates on a 50% increase in sCr was assessed using Cox proportional hazards models. All independent variables included in multivariate analyses were either categorical (coded as 0/1) or quantitative. Advanced age (≥ 65 years), endocapillary proliferative lesions (≥ 25%), crescentic lesions (≥ 25%), treatment with steroid pulse therapy and a RAS blockade were included as categorical variables. Systolic BP, amount of proteinuria, levels of sCr and serum albumin were included as quantitative variables. The results of the multivariate analyses are expressed as hazard ratios (HR), meaning ratios for a 50% increase sCr with a 95% confidence interval (CI). A p value of < 0.05 was considered significant for all data, which were statistically analyzed using IBM SPSS Advance Statistical Version 22.0.

## Results

### Clinicopathological findings and initial treatment at diagnosis

Table 1 compares the clinicopathological findings at diagnosis and initial treatment modalities between the adult and elderly groups. The mean age of the elderly group was 73.1 (range, 65 to 84) years. This group had a significantly higher systolic BP than the adult group, and 58.7% of them were hypertensive. The elderly patients also had significantly higher proteinuria values (2.91 vs. 2.35 g/day), worse renal function (sCr, 1.77 vs. 0.91 mg/dL; eGFR, 45.7 vs. 73.8 mL/min/1.73 m^2^), and lower serum albumin (3.05 vs. 3.55 g/dL) than the adult patients. Types of glomerulonephritis did not significantly differ between the groups. In contrast, the ratios of glomeruli with crescentic lesions were significantly higher in the elderly, than in the adult group (19.1% vs. 13.0%, p = 0.039).

**Table 1:** Comparison of clinicopathological findings at diagnosis and initial treatment between adult and elderly patients with IgAV-N (n = 152).

	Adults	Elderly	P
	(n = 106)	(n = 46)	
Age (y)	45.9 ± 14.4	73.1 ± 5.49	<0.001*
Gender (Male / Female)	45 / 61	26 / 20	0.110
**Clinical findings**			
Body mass index	23.5 ± 4.88	23.6 ± 4.06	0.881
Systolic BP (mmHg)	127.7 ± 16.5	139.4 ± 17.4	<0.001*
Diastolic BP (mmHg)	76.5 ± 11.0	77.3 ± 11.9	0.699
Hypertension at diagnosis	33 (31.1%)	27 (58.7%)	<0.001*
Sediment RBC > 30/HPF	57 (53.8%)	29 (63.0%)	0.721
Proteinuria (g/day)	2.35 ± 2.67	2.91 ± 2.92	0.250
UP > 3 g/day	24 (22.6%)	17 (37.0%)	0.068
Serum creatinine (mg/dL)	0.91 ± 0.46	1.77 ± 1.89	0.004*
Estimated GFR (mL/min/1.73 m^2^)	73.8 ± 27.6	45.7 ± 27.5	<0.001*
Serum albumin (g/dL)	3.55 ± 0.68	3.05 ± 0.60	<0.001*
Serum total cholesterol (mg/dL)	223.3 ± 62.7	204.2 ± 45.4	0.064
Diabetes mellitus at diagnosis	11 (10.4%)	9 (19.6%)	0.087
**Pathological findings**			
Mesangial proliferative GN	80 (75.5%)	26 (56.5%)	0.147
Endocapillary proliferative GN	7 (6.6%)	9 (19.6%)	
Crescentic and necrotizing GN	9 (8.5%)	5 (10.9%)	
Minor glomerular abnormality	4 (3.8%)	1 (2.2%)	
Focal segmental glomerulosclerosis	1 (0.9%)	0 (0.0%)	
Membranoproliferative GN	1 (0.9%)	1 (2.2%)	
Other	4 (3.8%)	4 (8.7%)	
Glomeruli with endocapillary lesions	18.7 ± 24.4	20.4 ± 26.3	0.711
Glomeruli with crescentic lesions	13.0 ± 15.1	19.1 ± 18.9	0.039*
**Initial treatment**			
Oral corticosteroid	84 (79.2%)	37 (80.4%)	0.752
Initial dose (mg/dL)	30.0 ± 14.5	27.3 ± 12.4	0.317
Pulse therapy	65 (61.3%)	23 (50.0%)	0.219
Courses of pulse therapy (n = 1 / 2 / 3)	23 / 15 / 27	9 / 5 / 9	
RAS blockades	59 (55.7%)	30 (65.2%)	0.231
Immunosuppressive agents	19 (17.9%)	7 (15.2%)	0.730
MMF / CPA / CyA / AZP	6 / 6 / 5 / 2	3 / 1 / 3 / 0	<0.001*
Tonsillectomy	34 (32.1%)	3 (6.5%)	0.001*

AZP, azathioprine; CPA, Cyclophosphamide; CyA, Cyclosporin A; GN, glomerulonephritis; MMF, Mycophenolate mofetil; UP, urinary protein. Data are shown as n (%) or means ± SD.

*p < 0.05, unpaired t test, chi-square test, or Fisher exact test.

Oral corticosteroid was the initial treatment for about 80% of the patients in both groups, with mean initial doses of 27.3 (elderly) and 30.0 (adult) mg/day. Although 61.3% and 50.0% of patients, respectively, in the adult and elderly groups received steroid pulse therapy, the number of courses varied between one and three. Over 50% of patients in both groups were initially treated with a RAS blockade. Significantly more adult than elderly patients underwent tonsillectomy (32.1% vs. 6.5%, p = 0.001). In contrast, < 20% of patients in both groups received immunosuppressive agents as initial therapy.

### Renal outcomes and onset of cardiovascular events

**Fig 2:**
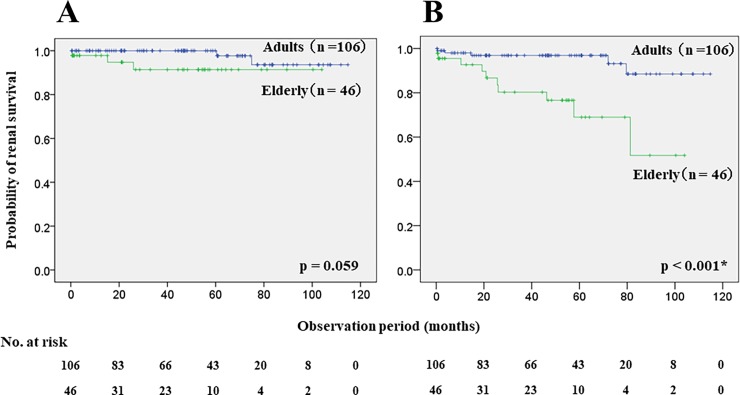
Kaplan-Meier analyses of renal outcomes between adult (n = 106) and elderly (n = 46) patients. (A) Nine-year renal survival rates for adult and elderly patients are 93.6% vs. 91.4% (p = 0.059, log-rank test). (B) Nine-year rates of freedom from 50% increase in serum creatinine from baseline between adult and elderly patients are 88.5% vs. 51.7% (p < 0.001, log-rank test).

Table 2 compares the clinical findings, renal outcomes and clinical events at the final observation between the two groups. Systolic BP, numbers of urinary red blood cells, and urinary protein concentrations were significantly higher in the elderly, than in the adult group. However, all findings were improved compared with those at diagnosis in both groups. Proteinuria was controlled at < 1.0 g/day in both groups, and 46.2% and 23.9% of the adult and elderly patients respectively, reached CR. Renal survival rates were favorable, with preservation of > 90% renal function in both groups during the nine-year observation period (93.6% vs. 91.4%, p = 0.059). In contrast, the rate of freedom from a 50% increase in sCr was significantly lower in the elderly, than in the adult group (51.7% vs. 88.5%, p < 0.001; Fig 2). As well as renal survival, significantly more elderly than adult patients had a 50% increase in sCr (21.7% vs. 4.7%, p = 0.002; Table 2). Four (8.7%) elderly and two (2.8%) adult patients died without statistical difference. The onset of cardiovascular diseases and malignancies also did not differ between the groups, although > 20% of the patients in both groups had diabetes mellitus.

**Table 2:** Comparison of clinical findings at final assessment, renal outcomes and clinical events between adult and elderly patients with IgAV-N (n = 152).

	Adults	Elderly	P
	(n = 106)	(n = 46)	
Follow-up period (m)	49.7 ± 31.2	37.9 ± 29.5	0.032*
**Clinical findings**			
Systolic BP (mmHg)	123.8 ± 16.1	131.7 ± 21.3	0.023*
Sediment RBC > 30/HPF	6 (5.7%)	8 (17.4%)	<0.001*
Proteinuria (g/day)	0.29 ± 0.42	0.77 ± 1.29	0.024*
UP > 1 g/day	7 (6.6%)	9 (19.6%)	0.017*
Disappearance of hematuria	64 (60.4%)	17 (37.0%)	0.015*
Disappearance of proteinuria	70 (66.0%)	25 (54.3%)	0.290
Clinical remission	49 (46.2%)	11 (23.9%)	0.016*
Serum creatinine (mg/dL)	0.98 ± 1.00	1.80 ± 2.11	0.116
Serum total protein (g/dL)	6.94 ± 0.54	6.63 ± 1.05	0.085
Serum total cholesterol (mg/dL)	188.6 ± 37.6	184.3 ± 35.7	0.567
**Outcomes and events**			
50% increase in sCr	5 (4.7%)	10 (21.7%)	0.002*
100% increase in sCr	3 (2.8%)	4 (8.7%)	0.200
ESKD with RRT	2(1.9%)	3 (6.5%)	0.163
Death	2 (1.9%)	4 (8.7%)	0.069
Cerebral infarction	1 (0.9%)	0 (0.0%)	0.697
Acute coronary syndrome	3 (2.8%)	0 (0.0%)	0.554
Malignancy	6 (5.7%)	4 (8.7%)	0.491
Diabetes mellitus	22 (20.8%)	10 (21.7%)	0.899

ESKD, end-stage kidney disease; RRT, renal replacement therapy; UP, urinary protein. Data are shown as n (%) or means ± SD.

*p < 0.05, un-paired t tests, chi-square tests or Fisher exact tests.

### Effect of treatment modalities on renal outcomes

**Fig 3:**
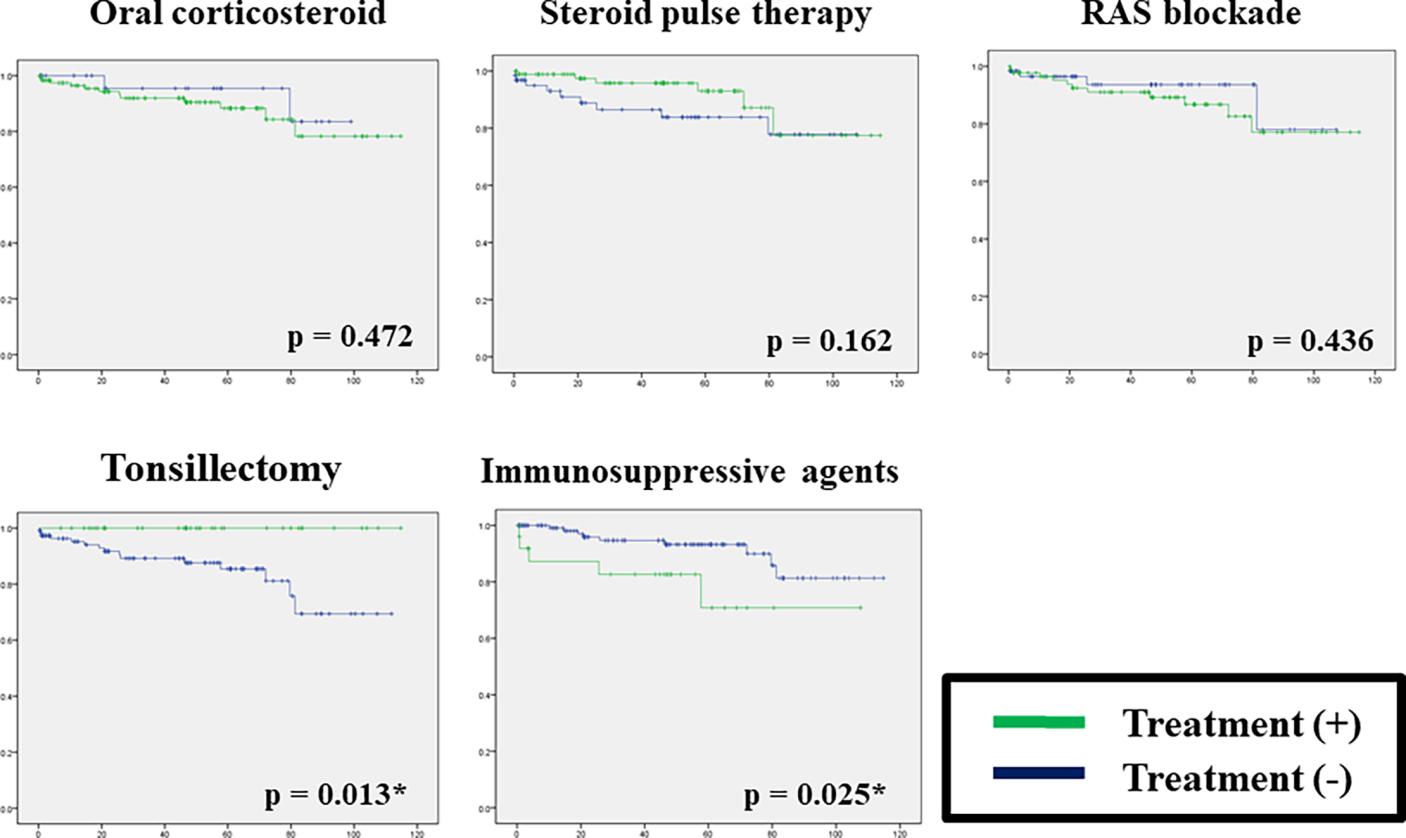
Time-to-event analyses of various initial treatment modalities. Kaplan-Meier curves for treated and untreated patients analyzed using log-rank tests. Endpoint was rates of freedom from 50% increase in serum creatinine from baseline.

Table 3 compares the clinical findings at diagnosis and initial treatment between patients with stable and decreased renal function at the final observation. Only serum albumin values significantly differed between groups with stable and deteriorating renal function (3.46 vs. 2.89 g/dL, respectively). The ratios of patients who were initially treated with steroid and a RAS blockade did not differ between the groups. On the other hand, all patients who underwent tonsillectomy had preserved stable renal function. The time-to-event findings determined from Kaplan-Meier curves and log-rank tests were the same. Therefore, tonsillectomy favorably affected renal function, whereas immunosuppressive agents significantly impaired renal outcome (Fig 3).

**Table 3:** Comparison of clinico-pathological findings at diagnosis and initial treatment between patients with stable and deteriorated renal function (n = 152).

	Renal function		
	Stable	Deteriorated	P
	(n = 137)	(n = 15)	
Age (y)	53.4 ± 17.4	60.9 ± 19.0	0.120
Gender (Male / Female)	63 / 74	8 / 7	0.599
**Clinical findings**			
Body mass index	23.5 ± 4.74	23.1 ± 3.73	0.717
Systolic BP (mmHg)	130.8 ± 17.5	136.4 ± 18.5	0.292
Diastolic BP (mmHg)	77.2 ± 11.2	72.8 ± 11.5	0.205
Sediment RBC >30/HPF	77 (56.2%)	9 (60.0%)	0.797
Proteinuria (g/day)	2.49 ± 2.79	2.71 ± 2.42	0.770
Serum creatinine (mg/dL)	1.14 ± 1.18	1.45 ± 1.14	0.333
Estimated GFR (mL/min/1.73 m^2^)	66.4 ± 30.1	55.4 ± 32.7	0.185
Serum albumin (g/dL)	3.46 ± 0.68	2.89 ± 0.60	0.002*
Serum total cholesterol (mg/dL)	217.4 ± 57.7	219.1 ± 68.3	0.916
**Pathological findings**			
Glomeruli with endocapillary lesions	18.5 ± 24.6	25.5 ± 27.2	0.300
Glomeruli with crescentic lesions	14.1 ± 15.9	21.7 ± 21.1	0.092
**Initial treatment**			
Oral corticosteroid	109 (79.6%)	12 (80.0%)	0.999
Steroid pulse therapy	82 (60.0%)	6 (40.0%)	0.207
RAS blockades	78 (56.9%)	11 (73.3%)	0.158
Immunosuppressive agents	21 (15.3%)	5 (33.3%)	0.073
Tonsillectomy	37 (27.0%)	0 (0.0%)	0.023*

Data are shown as n (%) or as means ± SD.

*p < 0.05, un-paired t test, chi-square test, or Fisher's exact test.

### Factors affecting a decline in renal function

The effects of the clinicopathological findings and initial treatment modalities on declining renal function (50% increase in sCr from baseline) were evaluated using multivariate analysis by Cox proportional hazards models. The likely prognostic factors for IgAV-N progression (age, BP, proteinuria, renal function, serum albumin, endocapillary proliferative and crescentic lesions) as well as steroid pulse therapy and RAS blockade were included as key independent variables in the model (Table 4). Univariate and multivariate analyses selected advanced age (≥ 65 years) and lower serum albumin values as significant independent prognostic factors affecting a decline in renal function in. In contrast, steroid pulse therapy in the multivariate analysis helped to prevent renal damage (HR, 0.25; 95% CI, 0.07–0.88, p = 0.031).

**Table 4:** Multivariate analysis of factors affecting the decline in renal function (n = 152).

Variable	Univariate analysis			Multivariate analysis		
	Hazard ratio	95% CI	P	Hazard ratio	95% CI	P
Elderly (> 65 vs. 19–64 y)	6.10	(2.07–18.0)	<0.001*	3.61	(1.09–12.0)	0.036*
Systolic BP (/mmHg)	1.02	(0.98–1.05)	0.308	0.99	(0.96–1.03)	0.895
Proteinuria (/g/day)	1.05	(0.88–1.24)	0.610	0.86	(0.68–1.11)	0.244
eGFR (/mL/min/1.73 m^2^)	0.99	(0.97–1.01)	0.151	1.01	(0.98–1.03)	0.514
Serum albumin (/0.1 g/dL)	0.24	(0.10–0.57)	<0.001*	0.20	(0.07–0.60)	0.004*
Endocapillary lesions (>25% vs. < 25%)	2.42	(0.87–6.72)	0.089	2.06	(0.57–7.47)	0.274
Crescentic lesions (>25% vs. < 25%)	1.93	(0.61–6.12)	0.266	3.07	(0.64–14.7)	0.160
Steroid pulse therapy (yes vs. no)	0.48	(0.17–1.37)	0.171	0.25	(0.07–0.88)	0.031*
RAS blockade (yes vs. no)	1.57	(0.50–4.95)	0.439	1.88	(0.53–6.63)	0.327

*Statistically significant.

## Discussion

The present cohort study elucidated that the elderly and low level of serum albumin were independent prognostic factors affecting the deterioration in renal function during observation. Furthermore, aggressive treatment intervention by corticosteroid and RAS blockade might contribute to good renal survival; especially steroid pulse therapy has a potential to prevent the progression of IgAV-N.

Whether clinical findings at diagnosis can predict long-term renal prognosis or not is still controversial in adult and elderly onset IgAV-N. Several cohort studies from Italy [11], France [12], and Finland [13] respectively showed that higher level of BP, large amount of proteinuria, and severe histological damage at diagnosis had possibility of concordant risk factors to decline in renal function. Mohey et al. confirmed that absolute renal risk (ARR) scores for IgAN comprising hypertension, proteinuria ≥ 1 g/day, and that severe pathological lesions were also useful for evaluating risk of IgAV-N [14]. By contrast, studies from Italy [15] and the UK [16] emphasized the importance of proteinuria during follow-up. These differences might have resulted from responses to initial therapies because the histological findings of IgAV-N often include acute reversible lesions such as endocapillary proliferation and crescentic formation as shown in the present study. In addition, IgAV-N might spontaneously resolve at a constant rate. Although higher BP and excessive proteinuria at diagnosis were not predictors in the present study, advanced age (≥ 65 years) and lower serum albumin were identified as prognostic factors. Pillebout et al. pointed out that advanced age (≥ 50 years) at onset was a powerful predictor of severe renal failure [12]. Rauta et al. notably found that lower serum albumin could predict IgAV-N progression in patients with normal renal function [13]. The present findings concur with those of Pillebout et al. and Rauta et al.

Information about renal prognosis is also limited. Previous reports indicate that the 10-year renal survival rates of patients with IgAV-N are about 68% in the UK [16], 75% in Italy [11], 80% in France [12], and 91% in Finland [13]. The nine-year renal survival rate in the present study was better than these, being 93.6% and 91.4% in adult and elderly patients, respectively. Of course the results of the present and these studies cannot be directly compared, because the severity of clinicopathological findings at baseline and the initial treatment modalities differed among them. Nonetheless, the present study found a favorable prognosis, especially for elderly patients, despite the severity of the clinical findings (60% of patients had hypertension, proteinuria 2.91 g/day and reduced renal function with eGFR of 45.7mL/min/1.73 m^2^ at diagnosis; Table 1). One reason for this might be because we aggressively treated even elderly patients with oral corticosteroid, intravenous steroid pulse therapy, and a RAS blockade.

Information about treatment strategies for adult and elderly patients with IgAV-N is presently scant. The Kidney Disease Improving Global Outcomes (KDIGO) guidelines suggest that IgAV-N in adults should be treated in the same manner as it is in children [19]. The recommended treatment for IgAV-N in children with persistent proteinuria > 1 g/day after a trial RAS blockade and GFR > 50 mL/min/1.73 m^2^ is the same as that for IgAN: a six-month course of corticosteroid. Meanwhile, concomitant steroid and cyclophosphamide is recommended for patients who have crescentic IgAV-N with nephrotic syndrome and/or deteriorating kidney function, with reference to crescentic IgAN [19]. The severity of IgAV-N in the elderly patients in this study seemed comparable to that of mild crescentic IgAV-N in children, and steroid pulse therapy favorably affected the prevention of progressive renal damage according to multivariate analysis. Niaudet et al. reported that of steroid pulse therapy preserves renal function in children with IgAV-N [23]. The French Vasculitis Study Group found a favorable response to corticosteroid therapy in 260 adult patients (including 144 who had undergone renal biopsies) [24]. Moreover, several case studies have found that corticosteroid and/or steroid pulse therapy can preserve renal function in elderly patients [25, 26]. In contrast, cyclophosphamide as an add-on to steroid does not confer a benefit compared with steroid alone [27]. Our univariate analysis also did not identify a positive contribution of immunosuppressive agents to the preservation of renal function (Fig 3).

Although a clinical trial would be desirable to resolve the lack of evidence described above, to survey the outcomes of rare renal disorders such as adult and elderly-onset IgAV-N is generally difficult. In fact, IgAV-N accounts for only 2.4% of all renal biopsies listed in the J-RBR between 2009 and 2010 [21]. The present multi-center study was relatively feasible due to having access to a large database. The J-RBR was the first nationwide, prospective registry of renal biopsies, and about 5,000 patients from 130 institutions in Japan have been registered annually since it was established in 2007 [20]. The registry contributes to not only the standardization of histological diagnoses and classification, but also to nationwide epidemiological studies of pathologies such as nephrotic syndrome and glomerulonephritis. Moreover, a secondary cohort study similar to the present investigation has been planned based on the J-RBR database for nephrotic syndrome in elderly patients [28, 29]. Secondary applications of the J-RBR database will become increasingly important in the future.

This study has some limitations. Firstly, the study design was retrospective with a mean observation period of only 3.9 years, because we used the J-RBR database established in 2007. We planned a multi-center study to increase the number of patients who were followed-up. Thus, the present study is one of the largest (n = 152) to investigate IgAV-N in adult and elderly patients but the sample size was not optimal. Although we tried to control confounding factors using multivariate analysis to evaluate prognostic factors and the effects of initial therapies, consideration for the comorbidities was not inadequate. Secondly, the number of items on the registration form was definitive, so we could not investigate the relationship between onset extra-renal symptoms such as purpura, arthritis, and gastrointestinal pain/bleeding and renal outcomes. We also could not evaluate the histological findings based on the Oxford classification and/or the International Society of Kidney Disease classification. Some studies have indicated that the Oxford Classification, which was originally designed for IgAN, can be used to evaluate the histological severity of IgAV-N [30, 31]. The present study examined only relationships between renal prognosis and endocapillary proliferative (E) and crescentic (C) lesions. The IgAN Classification Working Group recently proposed a revised Oxford Classification ‘MEST-C’ score that includes crescentic lesions [32]. Further studies are needed to validate the appropriateness and relevance of the proposed classification of IgAV-N including the significance of tubular atrophy/interstitial fibrosis and segmental glomerulosclerosis. Thirdly, we could not take detail information about the adverse effect of therapies. A recent major randomized controlled trial warned of the harmful effects of relatively high doses of oral methylprednisolone (0.6–0.8 mg/kg/day) on the onset of severe infections [33]. Although, scant evidence supports the notion that treatment with steroid, including intravenous pulse therapy, provokes severe adverse events in patients with IgAV-N, further verification is essential for safety. We could not evaluate the effects of tonsillectomy on the preservation of renal function using multivariate analyses, because all patients who underwent tonsillectomy had preserved, stable renal function without reaching the study outcomes. Notably, 32 (88.9%) of 36 patients who underwent tonsillectomy, were also treated with steroid pulse therapy, which significantly improves IgAN [34]. Moreover, this effect was also identified even in patients with recurrent IgAV-N after renal transplantation [35–37].

## Conclusions

The renal prognosis of adult and elderly patients with IgAV-N was favorable when aggressively treated with corticosteroid and a RAS blockade. However, the course of renal function should be carefully monitored in patients aged > 65 years and in those with hypoalbuminemia. Further studies of the mechanism of pathogenesis and histological findings are desirable to detail the characteristics of adult and elderly patients with IgAV-N. Additionally, an appropriately designed clinical trial is required to confirm the effects and safety of therapies with corticosteroid and a RAS blockade.

## Supporting information

S1 Table TREND checklist for the study. (PDF)

S1 File Study protocol submitting to Japan Society of Nephrology. (DOC)

S2 File Funding statement of the study. (DOCX)
