# Supplementary material for: Distinct characteristics and outcomes in elderly-onset IgA vasculitis (Henoch-Schönlein purpura) with nephritis: Nationwide cohort study of data from the Japan Renal Biopsy Registry (J-RBR)
Source: PLoS One. 2018 May 8;13(5):e0196955. doi: 10.1371/journal.pone.0196955 (PMC5940189; doi:10.1371/journal.pone.0196955)
Supplement: S1 File — (DOC) [file pone.0196955.s002.doc]

疫学研究

**成人紫斑病性腎症の予後に関する**

**観察研究（コホート研究）**

**研　究　実　施　計　画　書**

**日本腎臓学会腎疾患レジストリー**

**腎病理診断標準化委員会**

**公募研究に基づく二次研究**

**実施責任者 宮崎大学医学部　血液・血管先端医療学講座**

**氏名　藤元　昭一**

**主任研究者 宮崎大学医学部　医療人育成支援センター**

**宮崎大学医学部附属病院　第一内科（兼任）**

**氏名　　小松　弘幸**

**分担研究者 宮崎大学医学部附属病院　血液浄化療法部**

**氏名　　佐藤　祐二**

**宮崎大学医学部　内科学講座体液循環制御学**

**氏名　　福田　顕弘**

**作成日**

**2016年3月29日　計画書案　第1版作成**

**目　次**

　1．研究目的・・・・・・・・・・・・・・・・・・・・・・・・・・・・・・・・2

　2．研究概要・・・・・・・・・・・・・・・・・・・・・・・・・・・・・・・・2

　3．研究方法・・・・・・・・・・・・・・・・・・・・・・・・・・・・・・・・3

　4．研究実施場所・・・・・・・・・・・・・・・・・・・・・・・・・・・・・・5

　5．研究の対象患者の選定方法・・・・・・・・・・・・・・・・・・・・・・・・5

　6．対象数(目標症例数と統計解析方法)・・・・・・・・・・・・・・・・・・・・5

　7. 研究実施期間・・・・・・・・・・・・・・・・・・・・・・・・・・・・・・6

　8. 収集する資料及び試料・・・・・・・・・・・・・・・・・・・・・・・・・・6

9. 資料及び試料の保管及び破棄・・・・・・・・・・・・・・・・・・・・・・・6

10. 個人情報管理者の選定・・・・・・・・・・・・・・・・・・・・・・・・・・6

11. 個人の人権の擁護に関して・・・・・・・・・・・・・・・・・・・・・・・・6

12. 予想される対象者への利益および不利益(侵襲の程度、重篤な合併症、副作用)・・7

13. 不測の事態の場合の処置と補償・・・・・・・・・・・・・・・・・・・・・・7

14. 被験者の費用負担・・・・・・・・・・・・・・・・・・・・・・・・・・・・7

15. 研究資金および利益相反・・・・・・・・・・・・・・・・・・・・・・・・・8

16. 情報開示について・・・・・・・・・・・・・・・・・・・・・・・・・・・・8

17. 記録の保存と研究結果の公表・・・・・・・・・・・・・・・・・・・・・・・8

18. 目的外使用について・・・・・・・・・・・・・・・・・・・・・・・・・・・8

19. 参考資料・文献リスト・・・・・・・・・・・・・・・・・・・・・・・・・・8

**1．研究目的**

　日本最大規模の腎疾患データベースである腎生検レジストリー（Japan Renal Biopsy Registry: J-RBR）に登録された成人紫斑病性腎症例について、診断時の臨床病理学的所見と初期治療の内容、および腎予後（腎機能、尿所見）・生命予後を成人群（19～64歳）と高齢群（65歳以上）で比較検討し、これまで不明であった本邦の成人（特に高齢者）紫斑病性腎症の実態を明らかにすることを目的とする。

**2．研究概要**

**1) 研究の背景**

　紫斑病性腎炎（Henoch-Schönlein purpura nephritis: HSPN）は、IgA血管炎（2012年のChapel Hill Consensus Conferenceより名称変更）の30～60％にみられる腎病変であり、臨床的には血尿・蛋白尿あるいは腎機能障害を呈し、病理組織所見ではメサンギウム増殖を伴う糸球体腎炎所見とIgAの沈着を特徴とする^1)^。現時点で明確な発症機序は不明であるが、IgA腎症と同様に糖鎖不全IgA1の関与を示唆する報告も見られる^2),3)^。腎予後について、小児では一般に腎予後は良好とされてきた^4)^が、成人ではIgA腎症より良好とする報告^5)^や同等とする報告^6)-8)^があり、一致した見解はない。その要因として、成人の紫斑病性腎炎を対象とした臨床研究が少なく、対象症例数も少ないことなどが指摘されている。

　そのような背景のもと、我々は、日本腎臓学会主導で2007年より開始された腎生検レジストリー（Japan Renal Biopsy Registry: J-RBR）^9),10)^を用いた公募研究として、2007～2012年に登録されたIgA腎症5,679例と紫斑病性腎炎513例の臨床病理学的比較を行った。その結果、紫斑病性腎炎の年齢は0～19歳の小児と60～69歳の高齢層に2峰性のピークを有する分布をしていることが分かった^11)^。また、対象症例を、小児（≦18歳）、成人（19-64歳）、高齢（≧65歳）の3群に分けた場合、IgA腎症と紫斑病性腎炎の比較では、いずれの年齢層でもIgA腎症より紫斑病性腎炎の方が臨床病理学的重症度が有意に高いことが示された^11)^。さらに、紫斑病性腎炎513例について、上記の年齢別3群で比較した場合、高齢群は成人群や小児群と比較して、臨床症候で急速進行性腎炎症候群やネフローゼ症候群を呈する割合が高く、病理所見で管内増殖性糸球体腎炎や半月体形成性糸球体腎炎像を呈する割合が高く、臨床所見で高血圧や高度蛋白尿、腎機能低下、低アルブミン血症を呈する割合が高いことが示された^11)^。

　これらの解析結果から、高齢層の紫斑病性腎炎は発症頻度も高く、成人の紫斑病性腎炎や同年代のIgA腎症と比較しても診断時の臨床病理学的所見が重症であることが示され、その背景には、単なる加齢やそれに伴う動脈硬化性変化といった既知の因子だけでは説明が付かない、高齢発症の紫斑病性腎炎に特有の要因がある可能性も示唆された^11)^。

**2) 本研究の目的と目標**

　今回の我々の検討により、高齢発症の紫斑病性腎症において従来報告されていない診断時の特徴が明らかとなったが、これまで成人と高齢を区分した上で、その腎予後を検討された報告はほとんど見られない。さらに、腎予後に大きな影響を与える初期治療についても、現時点で十分なエビデンスに基づく治療法は確立しておらず、治療効果の検証についての検討は非常に限られているばかりか、実際どのような治療法が実施されているのかを十分な症例数で調査した研究自体が見られないのが現状である。

　そこで今回我々は、日本の大規模腎生検データベースであるJ-RBRの一次調査の結果^11)^に基づいて、成人および高齢症例の予後調査を実施し、両年齢群の腎および生命予後の違いを明らかにすることを本研究の第一目的とする。また、両群にどのような初期治療の介入がされていたか、あるいは治療に伴う副作用がどの程度みられたか、その実態を明らかにすることを第二目的とする。

　今回の検討の結果、成人および高齢発症紫斑病性腎炎症例への初期治療と予後の実態が明らかになるとともに、今後の新たな治療方法の検討や病態解明に有益な知見が得られると考える。

**3．研究方法**

（1）研究の種類・デザイン：観察研究（コホート研究）

（2）試験のアウトライン：

　①対象症例

- J-RBRに2007年7月～2012年末までに登録された19歳以上の症例を対象とする。
- 対象症例について、成人群（19～64歳）、高齢群（65歳以上）の2群を設定する。

　②2群間で比較する項目

- J-RBRに登録された患者基本情報（年齢/性別、臨床診断、臨床所見、病理組織所見など）
- 初期治療（ステロイド薬、レニン・アンジオテンシン系阻害薬、扁桃摘出術など）の内容
- 最終観察時の腎機能（血清クレアチニン値、末期腎不全の有無）および尿所見
- 観察期間中の重篤な合併症および死亡の有無

（3）症例登録の方法：

本研究では、2007年7月～2012年末までに既にJ-RBRへ登録された症例のみを解析対象とするため、本研究のための新たな症例登録および割付は行わない。

（4）被験者の研究参加予定期間：

本研究は、既にJ-RBRへ登録された症例の登録時データおよび最終観察時の診療録に基づくデータのみを解析対象とするため、本研究のための新たな被験者の研究参加はない。

（5）観察および検査項目

【J-RBRへの診断時登録データ】

　①患者基本情報：年齢、性別、臨床診断名、腎生検実施日、腎生検回数

　②一般所見：身長、体重、血圧（収縮期/拡張期）、降圧薬内服の有無、糖尿病診断の有無

　③尿所見：尿定性試験（尿潜血、尿蛋白）、尿沈渣（尿中赤血球数）、尿生化学（尿蛋白定量、g/日、g/gCr）

　④血液検査：血清クレアチニン（Cr）、血清総蛋白、血清アルブミン、総コレステロール、HbA1c

　⑤腎生検：病理組織診断名（IgA腎症または紫斑病性腎炎）

※診断時登録データについては、予め登録済みデータを記入したファイルを各施設に送付する。

【診断時の追加調査データ】

　①腎生検所見：採取糸球体数、管内増殖性病変を有する糸球体数、半月体形成を有する糸球体数

　②初期治療の内容：ステロイド薬、レニン・アンジオテンシン系阻害薬、扁桃摘出術、その他治療

【フォローアップ（最終観察時）のデータ】

①患者基本情報：年齢、最終診察日時

　②一般所見：血圧（収縮期/拡張期）、降圧薬内服の有無

　③尿所見：尿定性試験（尿潜血、尿蛋白）、尿沈渣（尿中赤血球数）、尿生化学（1日尿蛋白量、尿クレアチニン値）

　④血液検査：血清クレアチニン（Cr）、血清総蛋白、血清アルブミン、総コレステロール

　⑤合併症の有無：脳・心血管疾患（脳梗塞、脳出血、クモ膜下出血、急性冠症候群・心筋梗塞、大動脈解離、末梢血管疾患）、悪性腫瘍、心房細動、糖尿病、入院治療を要する感染症

（6）評価項目（エンドポイント）：

　①主要評価項目：腎機能（血清クレアチニン値の1.5および2倍化、末期腎不全の有無）

　②副次評価項目：死亡の有無、脳・心血管疾患の発生率、入院を要した合併症の発生率

（7）個々の被験者における中止基準：

本研究では、既にJ-RBRへ登録された症例データおよび診療録から得られたデータが解析対象となるため、本研究実施にあたって治療介入についての中止基準は該当しない。しかし、下記の状況が発生した場合には、研究を中止とする。

《中止基準》

1) 被験者が公示の説明ポスター等で本研究の実施を知り、研究参加の辞退の申し出があった場合

2) 本研究全体が中止された場合

3) その他の理由により、研究担当者が研究の中止が適当と判断した場合

（8）研究の進捗状況等の報告：

実施責任者は、毎年一回、研究の進捗状況を医学部長に報告する。

（9）研究実施計画書等の変更：

本研究の研究実施計画書や同意説明文書の変更または改訂を行う場合は、あらかじめ倫理委員会の承認を必要とする。

（10）研究の変更、中止・中断、終了：

　①研究の変更：本研究の研究実施計画書や同意説明文書の変更または改訂を行う場合は、あらかじめ倫理委員会の承認を必要とする。

　②研究の中止、中断：実施責任者は、審査委員会により中止の勧告あるいは指示があった場合は、研究を中止する。また、研究の中止または中断を決定した時は、速やかに医学部長にその理由とともに文書で報告する。

　③研究の終了：研究の終了時には、実施責任者は速やかに研究終了報告書を医学部長に提出する。

（11）研究実施体制

本研究は、日本腎臓学会腎疾患レジストリー腎病理診断標準化委員会の公募研究に基づく二次研究（コホート研究）であり、同委員会委員と共同研究者により運営委員会を設ける。

【運営委員会】

　金沢医科大学 腎臓内科 横山　仁

　岡山大学 腎臓内科 杉山　斉

　名古屋大学 腎臓内科 丸山彰一

　東北大学 臨床薬理学 佐藤　博

　群馬大学 腎臓内科 廣村桂樹

　熊本大学 腎臓内科 安藤政隆

　宮崎大学 腎臓内科　　 藤元昭一

　宮崎大学 腎臓内科 小松弘幸

【研究代表者】

　代表者名：藤元昭一（宮崎大学医学部　血液・血管先端医療学講座）

　事務局：宮崎大学医学部　血液・血管先端医療学講座事務

　　　　　TEL 0985-85-1510（内線2193）、FAX 0985-85-6596

E-mail: [fujimos@med.miyazaki-u.ac.jp](mailto:fujimos@med.miyazaki-u.ac.jp)

【共同研究施設および分担研究者名】

岩手県立中央病院 腎臓内科 相馬　淳

JCHO仙台病院 腎センター内科 佐藤壽伸

東北大学 臨床薬理学 佐藤　博

新潟大学 腎・膠原病内科 成田一衛

昭和大学 腎臓内科 柴田孝則

名古屋大学 腎臓内科 丸山彰一

藤田保健衛生大学 腎臓内科 湯澤由紀夫

金沢医科大学 腎臓内科 横山　仁

　岡山大学 腎臓内科 杉山　斉

　川崎医科大学 腎臓・高血圧内科 佐々木環

　九州大学 腎臓内科 鶴屋和彦

　久留米大学 腎臓内科 深水　圭

長崎大学 腎臓内科 西野友哉

熊本大学 腎臓内科 安藤政隆

　宮崎大学 腎臓内科　　 藤元昭一

【宮崎大学の研究体制】

　主任研究者：小松弘幸（宮崎大学医学部　医療人育成支援センター）

分担研究者：佐藤祐二（宮崎大学医学部附属病院　血液浄化療法部）

　　 ：福田顕弘（宮崎大学医学部内科学講座　循環体液制御学分野）

**4．研究実施場所**

　宮崎大学医学部附属病院第一内科（データ解析実施場所）

**5．研究の対象患者の選定方法**

（1）今回の研究対象者は、2007年7月から2012年末までにJ-RBRに登録された症例のうち、診断時の年齢が19歳以上であり、病理組織診断にて紫斑病性腎炎と診断された症例とする。これ以外の選択基準、除外基準は特に設けない。

**6．対象数（目標症例数と統計解析方法）**

（1）目標症例数とその設定根拠

　　成人紫斑病性腎炎：約180例

【設定根拠】

　2007年7月～2012年末までに紫斑病性腎症としてJ-RBRに登録された症例は、64施設より513例であった。このうち、18歳以下、19～64歳、65歳以上の症例数はそれぞれ、158例、259例、96例であった。19歳以上の登録355例について、登録数の多い上位20施設での登録合計数は238例であった。今回、この上位20施設の中から15施設程度との共同研究を想定しており、その際の対象症例数は最大で約180例と考えられる。

（2）統計解析方法

　①成人群（19～64歳）と高齢群（65歳以上）の背景因子の比較：

un-paired t testまたはMann-Whitney’s U testまたはχ2乗検定（Fisherの直接確率法を含む）

　②成人群と高齢群の初期治療内容の比較：

　　χ2乗検定（Fisherの直接確率法を含む）

　③成人群と高齢群の最終観察時の腎機能、尿所見等の比較：

un-paired t testまたはMann-Whitney’s U testまたはχ2乗検定（Fisherの直接確率法を含む）

　④成人群と高齢群の腎生存率の比較：

　　Kaplan-Meier法による生存解析（log-rank testによる検定）

　⑤成人群と高齢群の死亡の有無および脳・心血管疾患の発生率比較：

　　χ2乗検定（Fisherの直接確率法を含む）

　⑥紫斑病性腎症の腎予後に寄与する因子の解析：

　　Cox比例ハザードモデルによる多変量解析（ハザード比、95%信頼区間の解析）

**７．研究実施期間**

　倫理委員会承認後～2019年12月31日

**８．収集する資料及び試料**

　本研究での診断時データ収集は、既にJ-RBRへ症例登録された電子データのみを使用する。一方、予後解析のための最終観察時のデータ収集は、対象症例の診療録に記載されたデータのみとする。

また、本研究において新たに収集する試料はない。

**９．資料及び試料の保管及び破棄**

　本研究で収集した資料の保管は、以下の項にも示すとおり、個人情報管理者が厳重な個人情報管理のもとに、決められた場所で行う。今回の研究のために保管する試料（検体）はない。

**１０．個人情報管理者の選定**

　研究参加施設では、個人情報管理者（小松弘幸）を選定し、施設毎に個人情報の管理を行う。

**１１．個人の人権の擁護に関して**

　① 研究等の対象となる個人の人権の擁護

　　本研究は、「ヘルシンキ宣言」及び「人を対象とする医学系研究に関する倫理指針」を遵守して実施する。

　② 個人情報保護の体制

　　まず、本研究でアクセスするJ-RBR登録データは既に匿名化されており、各施設からの登録時に腎生検実施施設および施設番号のみが付与されている。本研究でのデータ解析時には、登録された各症例に暫定的な番号を付与することはあるが、個人の同定に至るような解析は一切行わない。また、J-RBRデータベースへのアクセス権は、解析を希望する研究が日本腎臓学会腎疾患レジストリー腎病理診断標準化委員会にて審議された後、アクセス権が承認された場合のみ付与される。そのアクセス権も研究申請者にのみ制限されている。本研究の解析も、この限定された情報アクセス条件の中で実施される。

　次に、各症例の最終観察時データ収集と管理については、個人情報管理者（小松弘幸）から各共同施設の研究協力者1名に対してのみ、既に匿名された状態でJ-RBR登録時に付与された「UMIN登録番号」および「症例登録番号」のみが示されたデータ記入用シートを送付し、そのシートに必要データを記入していただく。記入後は各施設協力者より個人情報管理者に記入シートを返送する。

収集および解析された結果を電子保存する場合は、ネットワークから切り離されたコンピュータに保存とし、ファイルにはパスワードを設定し、個人情報管理者（小松弘幸）が厳重に管理する。

研究の結果を公表する際は、被験者を特定できる情報を含まないようにする。

また、研究の目的以外に、研究で得られた被験者の試料等を使用しない。

　③研究等の対象患者に理解を求め同意を得る方法

　 本研究は、既に実施された診療結果（外来診療録や血液検査および尿検査データから得られる情報）に基づいて解析を行うものであり、研究実施期間中に対象患者には新たな侵襲は加わらない。したがって、本研究では、研究に対象者の診療録に記載された臨床データや血液検査、尿検査データを用いることを記した公示用の「説明ポスター」を作成し、研究参加施設の外来の掲示板に掲示することで周知を図り、本研究への参加を希望しない対象者の申し出を受け付けられるようにする。

　日本腎臓学会ホームページ上でも、本研究が実施されていることおよび本研究への参加施設を公開する。

**１２．予想される対象者への利益および不利益（侵襲の程度、重篤な合併症、副作用）**

（1）予想される利益：本研究への参加によって対象患者に直接の利益は生じない。しかし、本研究の成果により成人紫斑病性腎症の実態把握が可能となり、また、今後の治療法の確立に貢献できる可能性がある。

（2）予想される不利益（副作用）：本研究は既に登録されたデータを検証することが目的であり、対象患者への侵襲的かつ直接的な不利益は生じない。

**１３．不測の事態の場合の処置と補償**

　本研究は既に登録されたデータおよび診療時のデータを検証することが目的であり、本研究実施による治療上の不測の事態は発生しない。

**１４．本研究の費用負担**

　本研究は既にJ-RBRへ症例登録されたデータおよび診療時に得られたデータのみを解析対象とするため、患者のあらたな費用負担は発生しない。

**１５．研究資金および利益相反**

　本研究は実施責任者が所属する大学の大学運営費で実施する。また、本研究では利益相反は生じない。

**１６．情報開示について**

　本研究の登録患者から情報開示を求められた場合は、原則として、対象者に対して遅滞なく保有する情報を開示する。

**１７．記録の保存と研究結果の公表**

　実施責任者は、研究等の実施に係わる重要な文書（申請書類の控え、医学部長からの通知文書、各種申請書・報告書の控、その他データの信頼性を保証するのに必要な書類または記録等）を、研究の中止または終了後5年が経過した日までの間保存し、その後は個人情報に注意して廃棄する。研究担当者は、本研究の成果を関連学会等において発表することにより公表する。

**１８．目的外使用について**

　本研究では、既にJ-RBRへ症例登録されたデータおよび診療時の診療録記載データのみを解析対象とし、新たな試料（検体）の収集は行わないため、検体の目的外使用は生じない。

**１９．参考資料・文献リスト**

1. Rai A, Nast C, Adler S. Henoch-Schönlein purpura nephritis. J Am Soc Nephrol. 1999; 10: 2637-44.
2. Novak J, Moldoveanu Z, Renfrow MB, et. al. IgA nephropathy and Henöch-Schönlein purpura nephritis: Aberrant glycosylation of IgA1, formation of IgA1-containing immune complexes, and activation of mesangial cells. Contrib Nephrol. 2007; 157: 134-8.
3. Kiryluk K, Moldoveanu Z, Sanders JT, et. al. Aberrant glycosylation of IgA1 is inherited in both pediatric IgA nephropathy and Henoch-Schönlein purpura nephritis. Kidney Int. 2011; 80: 79-87.
4. Hung SP, Yang YH, Lin YT, et. al. Clinical manifestations and outcomes of Henoch-Schönlein purpura: comparison between adults and children. Pediatr Neonatol. 2009; 50: 162-8.
5. Calvo-Río V, Loricera J, Martín L, et. al. Henoch-Schönlein purpura nephritis and IgA nephropathy: a comparative clinical study. Clin Exp Rheumatol. 2013; 31: S45-S51.
6. Pillebout E, Thervet E, Hill G, Alberti C, Vanhille P, Nochy D. Henoch-Schönlein Purpura in adults: outcome and prognostic factors. J Am Soc Nephrol. 2002; 13: 1271-8.
7. Shrestha S, Sumingan N, Tan J, et. al. Henoch- Schönlein purpura with nephritis in adults: adverse prognostic indicators in a UK population. QJM. 2006; 99: 253-65.
8. Oh HJ, Ahn SV, Yoo DE, et. al. Clinical outcomes, when matched at presentation, do not vary between adult-onset Henöch-Schönlein purpura nephritis and IgA nephropathy. Kidney Int. 2012; 82: 1304-12.
9. Sugiyama H, Yokoyama H, Sato H, et. al.; Committee for Standardization of Renal Pathological Diagnosis and Working Group for Renal Biopsy Database, Japanese Society of Nephrology, Tokyo, Japan. Japan Renal Biopsy Registry: the first nationwide, web-based, and prospective registry system of renal biopsies in Japan. Clin Exp Nephrol. 2011; 15: 493-503.
10. Sugiyama H, Yokoyama H, Sato H, et. al.; Committee for Standardization of Renal Pathological Diagnosis; Committee for Kidney Disease Registry; Japanese Society of Nephrology. Japan Renal Biopsy Registry and Japan Kidney Disease Registry: Committee report for 2009 and 2010. Clin Exp Nephrol. 2013; 17: 155-173.
11. Komatsu H, Fujimoto S, Yoshikawa N, et. al. Clinical manifestations of Henoch-Schӧnlein purpura nephritis and IgA nephropathy: comparative analysis of data from the Japan Renal Biopsy Registry (J-RBR). Clin Exp Nephrol. 2015; (in press)
