# Supplementary material for: Distinct characteristics and outcomes in elderly-onset IgA vasculitis (Henoch-Schönlein purpura) with nephritis: Nationwide cohort study of data from the Japan Renal Biopsy Registry (J-RBR)
Source: PLoS One. 2018 May 8;13(5):e0196955. doi: 10.1371/journal.pone.0196955 (PMC5940189; doi:10.1371/journal.pone.0196955)
Supplement: S2 File — (DOCX) [file pone.0196955.s003.docx]

**Funding Statement**

This study was supported in part by the Japanese Society of Nephrology, by a Grant-in-Aid for Intractable Renal Diseases Research, Research on Rare and Intractable Diseases, and by Health and Labour Sciences Research Grants from the Ministry of Health, Labour and Welfare of Japan.
